# Maize yield prediction using machine learning: a systematic literature review

**DOI:** 10.3389/frai.2026.1735157

**Published:** 2026-04-20

**Authors:** Jabulani Nyengere, Frank Tchuwa, Harineck Mayamiko Tholo, Lucius Malalu, Allena Laura Njala, Petros Kachulu, Rodney Maganga, Brenda Matewere, Lackson Jamu, Clement Nyirenda, Jones Kanjira, Macdonald Chabwera, Patson Nalivata, Weston Mwase, Agness Mwangwela

**Affiliations:** 1 UKUDLA - African German Centre for Sustainable and Resilient Food Systems and Applied Agricultural and Food Data Science, Cape Town, South Africa; 2 Lilongwe University of Agriculture and Natural Resources, Lilongwe, Malawi; 3 Ndata School of Climate and Earth Sciences, Malawi University of Science and Technology, Limbe, Malawi; 4 Department of Computer Science, University of Western Cape (UWC), Bellville, South Africa

**Keywords:** data integration, food security, machine learning, maize yield prediction, remote sensing

## Abstract

**Introduction:**

Accurate maize yield prediction is critical for food security planning, particularly in sub-Saharan Africa, where maize is essential to national economies and livelihoods. This systematic review assesses the use of machine learning (ML) techniques in maize yield estimation, focusing on the methodologies, predictor variables, and results in peer-reviewed studies.

**Methods:**

The review followed the PRISMA 2021 guidelines, synthesizing 81 peer-reviewed studies published between 2014 and 2025. The analysis examined the ML algorithms, predictor variables, evaluation metrics, and methodological gaps identified in these studies.

**Results:**

The review found a significant increase in publications after 2021, reflecting growing confidence in the application of ML for agronomic decision-support. Random Forest (49.4%), XGBoost (16.1%), and Support Vector Machines (12.4%) were the most common algorithms, with hybrid deep-learning frameworks showing superior performance. Environmental variables, remote-sensing indices, and soil properties were the most frequently used predictors. RMSE and *R*^2^ were the primary evaluation metrics.

**Discussion:**

The findings underscore the challenges of data scarcity, limited interpretability, and geographical imbalance in the research, with Africa contributing less than 25% of the studies. There is a need for open-access agricultural data systems, hybrid explainable AI frameworks, and capacity building in computational agronomy to improve the effectiveness of ML applications in maize yield prediction.

## 1 Introduction

Maize (*Zea mays* L.) is one of the most important staple crops globally, particularly in sub-Saharan Africa, where it provides a major source of food, income, and livelihood security (Nyirenda et al., 2021). In Malawi, as in many other African countries, more than 80% of the population depends on maize as the primary staple crop, and both smallholder and commercial farmers rely heavily on its production for food security and economic stability (Stevens and Madani, 2016; Anghileri et al., 2024; Ng’ombe et al., 2024). Accurate and timely estimation of maize yield is therefore critical for planning at household, national, and regional levels (Chitsiko et al., 2022). Yield predictions inform agricultural policy, food reserve management, early warning systems, and resource allocation within agrifood systems, directly contributing to improved food and nutrition security (Bolton and Friedl, 2013).

Traditionally, maize yield estimation has relied on field surveys, farmer self-reporting, and statistical models that use limited biophysical and socio-economic data. While these approaches have been widely used, they often suffer from challenges such as data scarcity, subjectivity, high costs, and limited scalability (Li et al., 2022). Advances in remote sensing, big data, and computational technologies have enabled the emergence of modern approaches to yield prediction, particularly using machine learning (ML) algorithms (Jabed and Azmi Murad, 2024). According to Jhajharia et al. (2025), machine learning methods are data-driven, capable of handling large datasets with complex, non-linear relationships, and have demonstrated superior performance in various agricultural prediction tasks (Bolton and Friedl, 2013; Li et al., 2022). Several studies have highlighted the potential of ML techniques such as random forests, support vector machines, artificial neural networks, and deep learning architectures to improve the accuracy of yield estimation, particularly when integrated with remote sensing and climate datasets (Hamim et al., 2025; Jhajharia et al., 2025; Saha et al., 2025).

Despite significant advances in machine learning applications for crop yield prediction, the literature on maize yield forecasting in developing countries remains fragmented and unevenly developed (Muriuki et al., 2023; Omokpariola et al., 2025). Existing studies tend to concentrate on specific algorithms, individual data sources, or localized case studies, offering limited synthesis that directly responds to the needs of maize-dependent countries such as Malawi, where food security is highly sensitive to yield variability. Although broader reviews on crop modeling and yield forecasting are available, a comprehensive and systematic assessment focused specifically on machine-learning-based maize yield prediction in developing regions is still lacking. Such a synthesis is essential to consolidate evidence on methodological approaches, input variables, contextual challenges, and the comparative performance of machine learning algorithms across diverse agroecological settings (Dwivedi et al., 2019; van Klompenburg et al., 2020; Jabed and Azmi Murad, 2024). Accordingly, the present review focuses on maize-specific evidence and examines how algorithms, predictor variables, evaluation strategies, and implementation constraints vary across contexts relevant to developing countries.

The relevance of a systematic review in this field lies in its ability to critically assess existing research, identify methodological strengths and weaknesses, and map out knowledge gaps (Owens, 2021). For developing countries that face acute food security challenges, evidence-based recommendations on the applicability and reliability of machine learning methods are essential for guiding future research, investment, and policy (Gholami et al., 2022). Furthermore, synthesizing available studies can support the design of scalable, context-specific solutions that improve the resilience and sustainability of food systems. By highlighting key limitations in current approaches, such as data availability, model interpretability, or computational resource demands, a systematic review can also direct efforts toward addressing these constraints.

The present study therefore seeks to systematically review the application of machine learning models in maize yield prediction. Specifically, it aims to document the range of methods that have been used, assess the types of data and predictor variables incorporated, and evaluate the performance, challenges, and limitations of different approaches. The review also seeks to draw lessons and recommendations relevant to developing countries where maize is the primary staple food, with the goal of informing both research and practice. In doing so, this study contributes to ongoing discussions on leveraging machine learning to strengthen agricultural productivity, improve decision-making, and ensure sustainable food systems in the face of growing population pressure and climate variability.

The remainder of this paper is structured to present a logical progression of research. Section 2 details the methodology employed in this study. Subsequently, Section 3 presents the results, which are then discussed in detail in Section 4. Finally, Section 5 provides the conclusions drawn from the analysis and offers recommendations for future work.

## 2 Methodology

### 2.1 Review protocol

This systematic review was conducted following a rigorous protocol guided by the Preferred Reporting Items for Systematic Reviews and Meta-Analyses (PRISMA) statement, ensuring transparency and reproducibility using the updated guidelines for reporting systematic reviews provided by Page et al. (2021). The protocol was established at the outset to minimize bias and define the objectives, procedures, and criteria that would govern the entire process. Its formulation began with a focused research question on the scope of maize yield prediction using machine learning approaches, with specific attention to applications in developing countries. The review was executed in three interlinked stages: planning, conducting, and reporting.

#### 2.1.1 Planning stage

**Figure 1:**
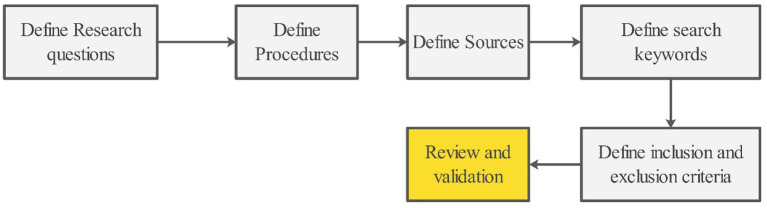
The plan review step by step process.

In the planning stage, the research questions were carefully articulated and refined to align with the review’s objectives. A comprehensive search strategy was developed and iteratively tested to ensure comprehensive retrieval of relevant literature. The protocol clearly defined the publication venues and selection criteria. Inclusion criteria specified that eligible studies must focus on maize yield prediction using machine learning, be published in English, and appear in peer-reviewed journals or conference proceedings. Conversely, exclusion criteria eliminated studies that did not directly address maize, relied solely on traditional (non-machine learning) methods, or lacked sufficient methodological detail. As in van Klompenburg et al. (2020), this stage concluded with an iterative review and validation of the entire protocol to ensure its feasibility and robustness (Figure 1).

#### 2.1.2 Conducting stage

**Figure 2:**
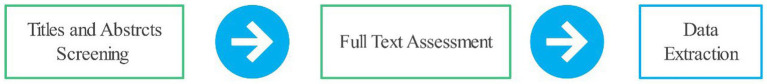
Three step screening stages.

Systematic searches were conducted across seven leading databases—ScienceDirect, Research4Life, Multidisciplinary Digital Publishing Institute (MDPI), Research gate, Institute of Electrical and Electronics Engineers (IEEE), Google Scholar and Springer Link—chosen for their extensive coverage of agricultural and computer science literature. The selection process followed a two-step approach: first, titles and abstracts were screened for relevance; subsequently, full-text articles were assessed against the predefined inclusion and exclusion criteria (Figure 2). To ensure reliability, the methodological quality of included studies was appraised using structured quality criteria, including clarity of objectives, transparency of methods, adequacy of data, and relevance to the research questions. Data extraction was performed using a structured template, capturing key details such as the author, publication year, study context, specific machine learning approaches, data features used for prediction, and performance outcomes. The extracted data were then synthesized both descriptively (e.g., publication year distribution, algorithm frequencies) and thematically (e.g., methodological strengths, research gaps).

#### 2.1.3 Reporting stage

The findings were documented and reported in accordance with PRISMA guidelines (Table 1). A PRISMA flow diagram was constructed to transparently illustrate the number of studies identified, screened, and included. Finally, the synthesized results were discussed in relation to the research questions, highlighting prevailing trends, comparative model performance, and critical gaps in the literature. Adherence to this structured protocol ensured a comprehensive and unbiased synthesis of the state of research on maize yield prediction. Study quality was assessed using a structured appraisal framework considering data source transparency, sample size adequacy, validation strategy, and reporting completeness. Studies with unclear validation procedures or insufficient methodological detail were retained for descriptive purposes but were not weighted heavily in performance comparisons. To minimize bias, findings were interpreted in light of spatial scale, dataset size, and validation design. ResearchGate was used only as a supplementary discovery platform to identify preprints or author-shared manuscripts, while inclusion decisions were restricted to peer-reviewed sources indexed in established databases.

**Table 1:** PRISMA summary table.

Stage of review process	Database/source	Records retrieved (n)	After duplicates removed (n)	Excluded (n)	Included (n)
Initial search	Science Direct	128			
	Research4Life	34			
	MDPI	62			
	Research gate	54			
	IEEE	5			
	Google Scholar	121			
	Springer Link	5			
Total records identified		409	387		
Records excluded after title/abstract screening				300	
Full-text articles assessed for eligibility					87
Full-text articles excluded (with reasons)				6	
Final studies included in the systematic review					81

### 2.2 Research questions

This systematic literature review (SLR) seeks to provide insights into the studies published in the field of machine learning and maize yield prediction. Studies have been analyzed from multiple dimensions to gain insight. This SLR study has established four research questions (RQs).

- RQ1—Which machine learning algorithms have been employed in the literature for maize yield prediction?
- RQ2—Which input features have been used in literature for maize yield prediction using machine learning?
- RQ3—Which evaluation parameters and evaluation approaches have been adopted in literature for maize yield prediction?
- RQ4—What key challenges exist in the field of maize yield prediction using machine learning?

### 2.3 Research strategy

The literature search was systematically structured to capture all studies relevant to the intersection of machine learning and maize prediction. Given the wide—ranging applications of machine learning, the search strategy was refined to ensure focus and relevance to the agricultural yield prediction domain. The initial exploratory search used keywords “machine learning” AND “yield prediction,” from which a preliminary pool of studies was retrieved. Abstracts were screened to identify synonyms and related terms that could expand the search coverage while maintaining relevance.

Subsequently, an automated, database-specific search was conducted using a more comprehensive Boolean expression designed to maximize retrieval of pertinent studies and minimizing omission of relevant literature. The final search string applied was: ((“machine learning” OR “artificial intelligence”) AND “data mining” AND (“yield prediction” OR “yield “forecasting” OR “yield estimation”)). This query was executed across seven databases: Science Direct, Research4Life, MDPI, Research gate, IEEE, Google Scholar and Springer Link.

A breakdown of the search strategy per database is as follows:

- Science Direct: The search string used is [“machine learning” AND “maize yield prediction”] (Title, Abstract, Keywords); and [((“machine learning” OR “artificial intelligence”) AND “data mining” AND (“maize yield prediction” OR “maize yield forecasting” OR “maize yield estimation”))] (Title, Abstract, Keywords).
- Research4Life: The search string used is the same query as above applied to (Title, Abstract, and Keywords).
- MDPI: The search string is [“machine learning” AND “maize yield prediction”] and [((“machine learning” OR “artificial intelligence”) AND “data mining” AND (“maize yield prediction” OR “maize yield forecasting” OR “maize yield estimation”))] (anywhere).
- Research gate: The search string is [“machine learning” AND “maize yield prediction”] (Anywhere).
- IEEE: The search string adopted is the same query as above applied in ResearchGate.
- Google Scholar: The search string is [((“machine learning” OR “artificial intelligence”) AND “data mining” AND (“maize yield prediction” OR “maize yield forecasting” OR “maize yield estimation”))] (anywhere).
- Springer Link: The search string is [((“machine learning” OR “artificial intelligence”) AND “data mining” AND (“maize yield prediction” OR “maize yield forecasting” OR “maize yield estimation”))] (anywhere).

### 2.4 Exclusion criteria

#### 2.4.1 Study selection and exclusion criteria

During the full-text assessment stage, articles that did not meet the predefined eligibility criteria were excluded, and the reasons for exclusion were recorded systematically. Common reasons included:

- Irrelevance to maize yield prediction—studies focusing on other crops or agricultural systems were excluded.
- Absence of machine learning techniques—papers that relied exclusively on traditional statistical, econometric, or biophysical modeling approaches without incorporating machine learning methods were excluded.
- Insufficient methodological detail—studies lacking transparency in data sources, model specification, or evaluation metrics, making reproducibility or meaningful interpretation impossible.
- Review papers, commentaries, or editorials—non-empirical works that did not contribute primary evidence.
- Language or accessibility limitations—studies not available in English or not accessible in full text.

After the initial screening process, the first two exclusion criteria were applied, resulting in a refined dataset of 87 studies eligible for further review. Subsequent application of all five exclusion criteria reduced the final selection to 81 studies that met the established inclusion standards and were retained for detailed analysis. Lastly, to address the four research questions, pertinent data were systematically extracted from each of the 81 included studies. The extracted information focused on determining whether each of the study satisfied the predefined inclusion requirements and contributed evidence to research questions. Studies that passed all exclusion criteria are cataloged in Appendix for transparency and reference.

## 3 Results

### 3.1 Distribution of research papers

A total of 409 research papers were initially retrieved across multiple databases and 81 studies met the eligibility criteria using the PRISMA guidelines (Table 2), Amongst the 81 papers, 23 were retained from Science Direct, 24 from MDPI, 20 from Google Scholar, 8 from Research4Life, 3 from ResearchGate, and 3 from Springer Link, while none from IEEE qualified for inclusion. This filtering process showed the increasing scientific rigor and relevance of publications in recent years as machine learning has gained momentum in agricultural analytics.

**Table 2:** Selection of papers based on the databases.

Database	Number of retrieved papers	Number of papers after exclusion criteria	Percentage of papers
Science Direct	128	23	28.40
Research4Life	34	8	9.87
MDPI	62	24	29.63
Research gate	54	3	3.70
IEEE	5	0	0
Google Scholar	121	20	24.7
Springer Link	4	3	3.70
Total	409	81	100

#### 3.1.1 Distribution of reviewed papers by year

**Figure 3:**
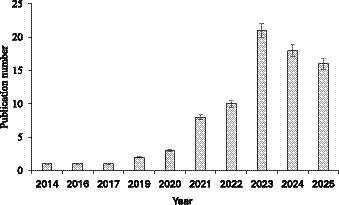
Distribution of the selected publications per year.

The temporal trend in maize yield prediction research (Figure 3) (Kheir et al., 2025b) demonstrates a significant increase in scholarly attention since 2021, following a period of modest publication output between 2014 and 2020. Early studies primarily explored traditional regression models, while later work increasingly incorporated advanced algorithms such as Random Forest (RF), XGBoost, and deep learning frameworks. The 2023 peak corresponds to the widespread adoption of high-resolution remote sensing technologies and hybrid machine learning models, which have enabled more accurate, scalable, and data-intensive yield prediction systems. This trend reflects a broader methodological shift toward data-driven precision agriculture, supported by advances in UAV imagery, multispectral satellite data, and ensemble learning frameworks (van Klompenburg et al., 2020; Saha et al., 2025; Zhang et al., 2025).

#### 3.1.2 Distribution of reviewed papers by country

**Figure 4:**
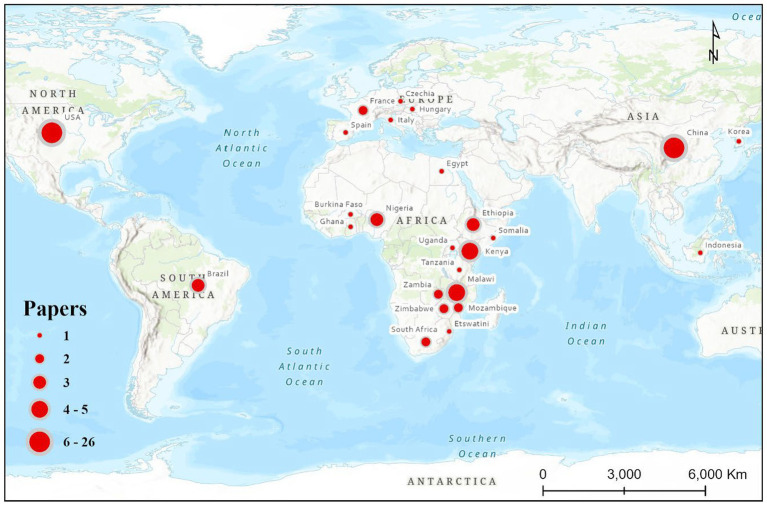
Research paper collection at country level.

As shown in Table 3, research productivity is geographically skewed toward technologically advanced regions. China (32.1%) and the United States (28.4%) dominate the field, driven by their robust digital agriculture infrastructure and strong investment in AI research. African representation remains comparatively limited, with Malawi contributing only four papers (4.9%), reflecting the continent’s ongoing challenges related to data availability, computational resources, and research funding. Notably, emerging contributions from Kenya, Ethiopia, Nigeria, and South Africa signal increasing regional engagement with ML-driven agronomic innovation. Figure 4 spatially illustrates this unequal distribution, revealing clusters of research intensity across Asia and North America, contrasted with sparse representation across sub-Saharan Africa.

**Table 3:** Research papers by country.

Country	Frequency
China	26
Malawi	4
Zimbabwe	2
Tanzania	1
Kenya	5
France	2
Somalia	1
Korea	1
South Africa	2
Etswatini	1
Brazil	3
Burkina Faso	1
Indonesia	1
USA	23
Uganda	1
Mozambique	2
Zambia	2
Ghana	1
Hungary	1
Italy	1
Nigeria	3
Ethiopia	3
Czechia	1
Egypt	1
Spain	1

#### 3.1.3 Distribution of reviewed papers by continent

When aggregated by continent (Table 4), Asia leads with 35.8% of the total studies, followed by America (30.9%), Africa (23.5%), and Europe (9.9%). The predominance of Asian and American studies likely reflects stronger digital-agriculture infrastructure, broader access to long time-series Earth-observation data and agricultural statistics, and greater investment in computational agronomy (Liakos et al., 2018; van Klompenburg et al., 2020). The growing African contribution, although still modest, is strategically important because recent studies from South Africa, southern Africa, Ghana, and East/Southern African regional analyses show increasing application of machine learning to maize forecasting under food-security and smallholder conditions (Adisa et al., 2019; Muthoni et al., 2021; Asamoah et al., 2024; Kenduiywo and Miller, 2024). This spatial distribution illustrates persistent disparities in research infrastructure and emphasizes the need for cross-continental data sharing and collaborative model development to broaden the use of ML in agriculture (Gholami et al., 2022).

**Table 4:** Research papers by continent.

Continent	Number
Africa	19
Asia	29
Europe	8
America	25

### 3.2 Summary of the studies reviewed

The final set of 81 studies (Table 5) covered a diverse range of methodologies, data sources, and prediction targets. Algorithms ranged from traditional statistical regressions to ensemble and deep-learning techniques. For example, Cheng et al. (2022) combined multi-indicator data with random forest regression and gradient boosting at county scale in China, Guo et al. (2023) compared RF, SVM, BP, PLSR, and LightGBM using UAV hyperspectral imagery, Muthoni et al. (2021) applied ML under conservation-agriculture conditions in southern Africa, and Taremwa et al. (2025) used a CNN-LSTM framework on multimodal climate and remote-sensing data in Uganda. Li et al. (2022) further demonstrated the relevance of satellite data for smallholder intercropped systems in Malawi. Collectively, these studies indicate a methodological evolution from single-source, experimental models toward increasingly multimodal and operational yield-forecasting workflows.

**Table 5:** Final studies included in the systematic review.

Retrieved from	Reference	Title	Year	Algorithm used	Complete bibliography
Google Scholar	S. García-Cortés et al.	A machine learning approach for estimating forage maize yield and quality in NW Spain	2025	LightGBM models	García-Cortés, S., Menéndez-Díaz, A., Bande-Castro, M. J., Carballal-Samalea, A., Martínez-Fernández, A., and Oliveira-Prendes, J. A. (2025). A machine learning approach for estimating forage maize yield and quality in NW Spain. PLOS ONE, 20(8), e0326364. https://doi.org/10.1371/journal.pone.0326364
Springer	A. Nyéki et al.	Application of spatio-temporal data in site-specific maize yield prediction with machine learning methods	2021	Extreme gradient boosting (XGBoost)	Nyéki, A., Kerepesi, C., Daróczy, B., Benczúr, A., Milics, G., Nagy, J., Harsányi, E., Kovács, A. J., and Neményi, M. (2021). Application of spatio-temporal data in site-specific maize yield prediction with machine learning methods. Precision Agriculture, 22, 1397–1415. https://doi.org/10.1007/s11119-021-09833-8
Research4life	K. Dilmurat et al.	AI-driven maize yield forecasting using unmanned aerial vehicle-based hyperspectral and LiDAR data fusion	2022	H_2_O Automated Machine Learning framework (H2O-AutoML)	Dilmurat, K., Sagan, V., and Moose, S. (2022). AI-driven maize yield forecasting using unmanned aerial vehicle-based hyperspectral and LiDAR data fusion. ISPRS Annals of the Photogrammetry, Remote Sensing and Spatial Information Sciences, V-3-2022, 193–199. https://doi.org/10.5194/isprs-annals-V-3-2022-193-2022
Research4life	T. Chigwada et al.	Maize Crop Yield Prediction Model Using Machine Learning	2023	Random Forest Regressor (RFR)	Chigwada, T., Dzinomwa, M., Sibanda, K., Moyo, S., and Ndlovu, B. (2023). Maize crop yield prediction model using machine learning. In Proceedings of the 4th Asia Pacific International Conference on Industrial Engineering and Operations Management. https://doi.org/10.46254/AP04.20230046
Research4life	F. Babaie Sarijaloo, M. Porta, B. Taslimi et al.	Yield performance estimation of corn hybrids using machine learning algorithms	2021	Extreme gradient boosting (XGBoost)	Babaie Sarijaloo, F., Porta, M., Taslimi, B., and Pardalos, P. M. (2021). Yield performance estimation of corn hybrids using machine learning algorithms. Artificial Intelligence in Agriculture, 5, 82–89. https://doi.org/10.1016/j.aiia.2021.05.001
Science Direct	T. C. Olayinka et al.	A data-driven machine learning approach toward an improved maize crop production	2025	Naïve Bayes, SVM, KNN, Decision Trees, ANN, and an innovative hybridized ANN-KNN classifier	Olayinka, T. C., Adetunmbi, A. O., Obe, O. O., Ibam, E. O., and Olayinka, A. S. (2025). A data-driven machine learning approach toward an improved maize crop production. Franklin Open, 12, 100334. https://doi.org/10.1016/j.fraope.2025.100334
Science Direct	A. P. Marques Ramos, et al.	A random forest ranking approach to predict yield in maize with uav-based vegetation spectral indices	2020	Random Forest Ranking	Marques Ramos, A. P., Prado Osco, L., Elis Garcia Furuya, D., et al. (2020). A random forest ranking approach to predict yield in maize with UAV-based vegetation spectral indices. Computers and Electronics in Agriculture, 178, 105791. https://doi.org/10.1016/j.compag.2020.105791
Science Direct	M. Cheng et al.	Combining multi-indicators with machine-learning algorithms for maize yield early prediction at the county-level in China	2022	random forest regression (RFR) and gradient boosting decision tree (GBDT)	Cheng, M., Penuelas, J., McCabe, M. F., Atzberger, C., Jiao, X., Wu, W., and Jin, X. (2022). Combining multi-indicators with machine-learning algorithms for maize yield early prediction at the county-level in China. Agricultural and Forest Meteorology, 323, 109,057. https://doi.org/10.1016/j.agrformet.2022.109057
Science Direct	Y. Guo et al.	Comparison of different machine learning algorithms for predicting maize grain yield using UAV-based hyperspectral images	2023	backpropagation neural network (BP), random forest regression (RF), support vector machine (SVM), partial least squares regression (PLSR), Light Gradient Boosting Machine (LightGBM)	Guo, Y., Xiao, Y., Hao, F., Zhang, X., Chen, J., de Beurs, K., He, Y., and Fu, Y. H. (2023). Comparison of different machine learning algorithms for predicting maize grain yield using UAV-based hyperspectral images. International Journal of Applied Earth Observation and Geoinformation, 124, 103528. https://doi.org/10.1016/j.jag.2023.103528
Science Direct	D. Lee et al.	Maize yield forecasts for Sub-Saharan Africa using Earth Observation data and machine learning	2022	Extremely randomized trees (ERT) model	Lee, D., Davenport, F., Shukla, S., Husak, G., Funk, C., Harrison, L., McNally, A., Rowland, J., Budde, M., and Verdin, J. (2022). Maize yield forecasts for Sub-Saharan Africa using Earth observation data and machine learning. Global Food Security, 33, 100643. https://doi.org/10.1016/j.gfs.2022.100643
Research gate	Sitienei et al.; Asian J. Prob. Stat., vol. 24, no. 1, pp. 1–9, 2023; Article no. AJPAS.103424	An Application of K-Nearest-Neighbor Regression in Maize Yield Prediction	2023	XGBOOST regression algorithm	Sitienei, M., Otieno, A., and Anapapa, A. (2023). An application of K-nearest-neighbor regression in maize yield prediction. Asian Journal of Probability and Statistics, 24(4), 1–10. https://doi.org/10.9734/ajpas/2023/v24i4529
Research gate	K. Halder et al.	High-Resolution Maize Yield Mapping across Africa using Earth Observation and Machine Learning, Deep Learning, and Foundation Model	2025	XGBoost, LightGBM, a hybrid deep neural network	Halder, K., Ewert, F.and Srivastava, A. K. (2026). High-resolution maize yield mapping across Africa using earth observation and machine learning, deep learning, and foundation model. Science of Remote Sensing, 13, 100344. https://doi.org/10.1016/j.srs.2025.100344
MDPI	O. M. Adisa et al.	Application of Artificial Neural Network for Predicting Maize Production in South Africa	2019	Artificial Neural Network	Adisa, O. M., Botai, J. O., Adeola, A. M., Hassen, A., Botai, C. M., Darkey, D., and Tesfamariam, E. (2019). Application of artificial neural network for predicting maize production in South Africa. Sustainability, 11(4), 1145. https://doi.org/10.3390/su11041145
Google Scholar	Taremwa et al.	Prediction of Maize Yield in Uganda using CNNLSTM Architecture on a Multimodal Climate and Remote Sensing Dataset	2025	Convolutional neural network and long short-term memory (CNN-LSTM)	Taremwa, D., Ahishakiye, E., Obbo, A. et al. Prediction of maize yield in Uganda using CNN-LSTM architecture on a multimodal climate and remote sensing dataset. Discov Artif Intell 6, 164 (2026). https://doi.org/10.1007/s44163-026-00855-7
Science Direct	B. K. Kenduiywo and S. Miller et al.	Seasonal Maize yield forecasting in South and East African Countries using hybrid Earth observation models	2024	Random Forest (RF)	Kenduiywo, B. K., and Miller, S. (2024). Seasonal maize yield forecasting in South and East African countries using hybrid Earth observation models. Heliyon, 10(13), e33449. https://doi.org/10.1016/j.heliyon.2024.e33449
Science Direct	E. Asamoah et al.	Random forest machine learning for maize yield and agronomic efficiency prediction in Ghana	2024	Random forest	Asamoah, E., Heuvelink, G. B. M., Chairi, I., Bindraban, P. S., and Logah, V. (2024). Random forest machine learning for maize yield and agronomic efficiency prediction in Ghana. Heliyon, 10(17), e37065. https://doi.org/10.1016/j.heliyon.2024.e37065
Google Scholar	Bao et al.	Forecasting spring maize yield using vegetation indices and crop phenology metrics from UAV observations	2023	Gradient-boosted regression tree (GBRT)	Bao, L., Li, X., Yu, J., Li, G., Chang, X., Yu, L., and Li, Y. (2024). Forecasting spring maize yield using vegetation indices and crop phenology metrics from UAV observations. Food and Energy Security, 13, e505. https://doi.org/10.1002/fes3.505
Science Direct	O. T. Faloye et al.	Forecasting maize yield from growth parameters using machine learning in a biochar-inorganic fertilizer amended soil under drip irrigation	2025	Support Vector Machine (SVM), Artificial Neural Network (ANN), and Boosted Trees (BT)	Faloye, O. T., Ajayi, A. E., Kamchoom, V., Sinsamutpadung, N., Adeyeri, O., and Ogunwole, J. O. (2025). Forecasting maize yield from growth parameters using machine learning in a biochar-inorganic fertilizer amended soil under drip irrigation. Smart Agricultural Technology, 13, 101490. https://doi.org/10.1016/j.atech.2025.101490
Google Scholar	Fashoto et al., Malaysian Journal of Computing, (Adisa et al., 2019): 679–697, 2021	Implementation of machine learning for predicting maize crop yields using multiple linear regression and backward elimination.	2021	multiple linear regression	Fashoto, S., Mbunge, E., Ogunleye, G., and van den Burg, J. (2021). Implementation of machine learning for predicting maize crop yields using multiple linear regression and backward elimination. Malaysian Journal of Computing, 6(1), 679–697. https://doi.org/10.24191/mjoc.v6i1.8822
Google Scholar	O. M. Olanrewaju et al.	Intelligent Maize Yield Prediction Model Based on Plant Attributes and Machine Learning Algorithms	2024	Random Tree, Random Forest and Neural Networks.	Olanrewaju, Oyenike and Jiya, Eli and Echobu, Faith. (2024). Intelligent Maize Yield Prediction Model Based on Plant Attributes and Machine Learning Algorithms. International Journal of Research and Scientific Innovation. XI. 1097–1104. 10.51244/IJRSI.2024.1107087. https://doi.org/10.51244/IJRSI.2024.1107087
Google Scholar	F. Muthoni et al.	Machine learning model accurately predict maize grain yields in conservation agriculture systems in Southern Africa	2021	Random Forest	F. Muthoni, C. Thierfelder, B. Mudereri, J. Manda, M. Bekunda and I. Hoeschle-Zeledon, “Machine learning model accurately predict maize grain yields in conservation agriculture systems in Southern Africa,” 2021 9th International Conference on Agro-Geoinformatics (Agro-Geoinformatics), Shenzhen, China, 2021, pp. 1–5, doi: 10.1109/Agro-Geoinformatics50104.2021.953033
Google Scholar	W. Mupangwa et al.	Evaluating machine learning algorithms for predicting maize yield under conservation agriculture in Eastern and Southern Africa	2020	Linear Algorithms	Mupangwa, W., Chipindu, L., Nyagumbo, I., Mkuhlani, S., and Sisito, G. (2020). Evaluating machine learning algorithms for predicting maize yield under conservation agriculture in Eastern and Southern Africa. SN Applied Sciences, 2, 952. https://doi.org/10.1007/s42452-020-2711-6
Google Scholar	P. V. D. Souza et al.	Maize Yield Prediction using Artificial Neural Networks based on a Trial Network Dataset	2023	Artificial Neural Network (ANN)	Duarte de Souza, P. V., Rezende, L. P., Duarte, A. P., and Miranda, G. V. (2023). Maize yield prediction using artificial neural networks based on a trial network dataset. Engineering, Technology and Applied Science Research, 13(2), 10,338–10,346. https://doi.org/10.48084/etasr.5664
Google Scholar	Wang et al.	Maize yield prediction with trait-missing data via bipartite graph neural network	2024	Bipartite Graph Neural Network	Wang, K., Han, Y., Zhang, Y., Zhang, Y., Wang, S., Yang, F., Liu, C., Zhang, D., Lu, T., Zhang, L., and Liu, Z. (2024). Maize yield prediction with trait-missing data via bipartite graph neural network. Frontiers in Plant Science, 15, 1433552. https://doi.org/10.3389/fpls.2024.1433552
MDPI	Z. Mondschein et al.	Mapping Field-Level Maize Yields in Ethiopian Smallholder Systems Using Sentinel-2 Imagery	2024	Random forest regression	Mondschein, Z., Paliwal, A., Sida, T. S., Chamberlin, J., Wang, R., and Jain, M. (2024). Mapping field-level maize yields in Ethiopian smallholder systems using Sentinel-2 imagery. Remote Sensing, 16(18), 3451. https://doi.org/10.3390/rs16183451
Science Direct	Z. Guo et al.	Smallholder maize yield estimation using satellite data and machine learning in Ethiopia	2023	Linear regression, Support vector machine, Regression trees, Gaussian process regression, Random forest regression, Neural networks	Guo, Z., Chamberlin, J., and You, L. (2023). Smallholder maize yield estimation using satellite data and machine learning in Ethiopia. Crop and Environment, 2(10), 100? [journal metadata should be spot-checked]. https://doi.org/10.1016/j.crope.2023.07.002
Science Direct	H. Zhang et al.	Optimizing corn yield prediction: Integrating multi-temporal UAS data and machine learning	2025	LASSO regression, Random forest regression, Gradient boosting regression	Zhang, H., Zhou, Y., Ma, S., and Yemoto, K. (2025). Optimizing corn yield prediction: Integrating multi-temporal UAS data and machine learning. Smart Agricultural Technology, 12, 101344. https://doi.org/10.1016/j.atech.2025.101344
Research gate	H. R. Seireg et al.	Cascading Ensemble Machine Learning Algorithms for Maize Yield Level Prediction	2023	K Nearest Neighbors (KNN), Naive Bayes (NB), Decision Tree Classifier (DTC), Quadratic Discriminant Analysis (QDA)	Seireg, H. R., Omar, Y. M., El-Sayed, F., El-Fishawy, A. S., and Elmahalawy, A. (2023). Cascading ensemble machine learning algorithms for maize yield level prediction. Menoufia Journal of Electronic Engineering Research, 32(2), 1–13. https://doi.org/10.21608/mjeer.2023.159995.1066
Research4life	VAFAEINEJAD et al.	Robust County-Level Corn Yield Estimation Using Ensemble Machine Learning and Multisource Remote Sensing	2025	Random forest (RF) and extreme gradient boosting (XGBoost)	A. Vafaeinejad, A. Sharifi and S. N. Khan, “Robust County-Level Corn Yield Estimation Using Ensemble Machine Learning and Multisource Remote Sensing,” in IEEE Journal of Selected Topics in Applied Earth Observations and Remote Sensing, vol. 18, pp. 16942–16953, 2025, doi:10.1109/JSTARS.2025.3585779.
MDPI	C. D. Villiers et al.	Assessing Maize Yield Spatiotemporal Variability Using Unmanned Aerial Vehicles and Machine Learning	2024	Random Forest, Gradient Boosting (GradBoost), Categorical Boosting, and Extreme Gradient Boosting	de Villiers, C., Mashaba-Munghemezulu, Z., Munghemezulu, C., Chirima, G. J., and Tesfamichael, S. G. (2024). Assessing maize yield spatiotemporal variability using unmanned aerial vehicles and machine learning. Geomatics, 4(3), 213–236. https://doi.org/10.3390/geomatics4030012
MDPI	D. Radoˇcaj et al.	Phenology-Based Maize and Soybean Yield Potential Prediction Using Machine Learning and Sentinel-2 Imagery Time-Series	2025	Random forest (RF), support vector machine regression (SVM), multivariate adaptive regression splines (MARS), and Bayesian regularized neural networks (BRNNs)	Radočaj, D., Plaščak, I., and Jurišić, M. (2025). Phenology-based maize and soybean yield potential prediction using machine learning and Sentinel-2 imagery time-series. Applied Sciences, 15(13), 7216. https://doi.org/10.3390/app15137216
Google Scholar	Sitienei et al.	Random Forest Regression in Maize Yield Prediction	2023	Random Forest Regression	Sitienei, M., Anapapa, A., and Otieno, A. (2023). Random Forest Regression in Maize Yield Prediction. Asian Journal of Probability and Statistics, 23(4), 43–52. https://doi.org/10.9734/ajpas/2023/v23i4511
Google Scholar	M. Shahhosseini et al.	Maize yield and nitrate loss prediction with machine learning algorithms	2019	LASSO Regression, Ridge Regression, random forests, Extreme Gradient Boosting	Shahhosseini, M., Martinez-Feria, R., Hu, G., and Archontoulis, S. (2019). Maize yield and nitrate loss prediction with machine learning algorithms. Environmental Research Letters, 14, 124,026. https://doi.org/10.1088/1748-9326/ab5268.
Google Scholar	Y. Chang et al.	A data-driven crop model for maize yield prediction	2023	Linear regression	Chang, Y., Latham, J., Licht, M., and Wang, L. (2023). A data-driven crop model for maize yield prediction. Communications Biology, 6, 439. https://doi.org/10.1038/s42003-023-04833-y
Google Scholar	X. Li et al.	Maize yield estimation in Northeast China’s black soil region using a deep learning model with attention mechanism and remote sensing	2025	Multi-layer perceptron (MLP), support vector machines (SVMs), and random forests (RFs)	Li, X., Lyu, Y., Zhu, B., and Zhang, Y. (2025). Maize yield estimation in Northeast China’s black soil region using a deep learning model with attention mechanism and remote sensing. Scientific Reports, 15, 12927. https://doi.org/10.1038/s41598-025-97563-6
MDPI	Y. Yu et al.	Research on Mass Prediction of Maize Kernel Based on Machine Vision and Machine Learning Algorithm	2025	Partial Least Squares Regression (PLSR), Random Forest (RF), Support Vector Regression (SVR), and K-Nearest Neighbors (KNN)	Yu, Y., Fan, C., Li, Q., Wu, Q., Cheng, Y., Zhou, X., He, T., and Li, H. (2025). Research on Mass Prediction of Maize Kernel Based on Machine Vision and Machine Learning Algorithm. Processes, 13(2), 346. https://doi.org/10.3390/pr13020346.
MDPI	M. Croci et al.	Dynamic Maize Yield Predictions Using Machine Learning on Multi-Source Data	2023	Gaussian process regression (GPR), Principal Component Analysis (PCA)	Croci, M., Impollonia, G., Meroni, M., and Amaducci, S. (2023). Dynamic Maize Yield Predictions Using Machine Learning on Multi-Source Data. Remote Sensing, 15(1), 100. https://doi.org/10.3390/rs15010100
Research4life	Ko et al.	Assessing maize growth and yield using a remote sensing–integrated crop model enhanced with lasso and ridge regression	2025	lasso and ridge regression	Ko, J., Shin, T., Ban, J., and Kim, H. Y. (2025). Assessing maize growth and yield using a remote sensing–integrated crop model enhanced with lasso and ridge regression. Journal of Applied Remote Sensing, 19(2), 028506. https://doi.org/10.1117/1.JRS.19.028506
Research4life	S. N. KHAN ET AL.	County-level corn yield prediction using supervised machine learning	2023	PLSR, support vector regression (SVR) and ridge regression	Khan, S. N., Khan, A. N., Tariq, A., Lu, L., Malik, N. A., Umair, M., Hatamleh, W. A., and Zawaideh, F. H. (2023). County-level corn yield prediction using supervised machine learning. European Journal of Remote Sensing, 56(1), 2253985. https://doi.org/10.1080/22797254.2023.2253985
MDPI	S. Sarkar et al.	Integrating Remote Sensing and Soil Features for Enhanced Machine Learning-Based Corn Yield Prediction in the Southern US	2025	Multiple linear regression (MLR), RF, XGBoost, and GBR	Sarkar, S., Osorio Leyton, J. M., Noa-Yarasca, E., Adhikari, K., Hajda, C. B., and Smith, D. R. (2025). Integrating Remote Sensing and Soil Features for Enhanced Machine Learning-Based Corn Yield Prediction in the Southern US. Sensors, 25(2), 543. https://doi.org/10.3390/s25020543
MDPI	F. H. R. Baio et al.	Maize Yield Prediction with Machine Learning, Spectral Variables and Irrigation Management	2022	Artificial Neural Network (ANN), M5P Decision Tree (J48), REPTree Decision Tree (REPT), Random Forest (RF), and Support Vector Machine (SVM)	Baio, F. H. R., Santana, D. C., Teodoro, L. P. R., de Oliveira, I. C., Gava, R., de Oliveira, J. L. G., da Silva Junior, C. A., Teodoro, P. E., and Shiratsuchi, L. S. (2022). Maize yield prediction with machine learning, spectral variables and irrigation management. Remote Sensing, 15(1), 79. https://doi.org/10.3390/rs15010079
MDPI	Z. Ji et al.	Prediction of Corn Yield in the USA Corn Belt Using Satellite Data and Machine Learning: From an Evapotranspiration Perspective	2022	LASSO, SVR, RF and LSTM.	Ji, Z., Pan, Y., Zhu, X., Zhang, D., and Dai, J. (2022). Prediction of corn yield in the USA Corn Belt using satellite data and machine learning: From an evapotranspiration perspective. Agriculture, 12(8), 1263. https://doi.org/10.3390/agriculture12081263
MDPI	M. F. d. Oliveira et al.	Training Machine Learning Algorithms Using Remote Sensing and Topographic Indices for Corn Yield Prediction	2022	Extremely randomized trees, gradient boosting machine (GBM), XGBoost algorithms	de Oliveira, M. F., Ortiz, B. V., Morata, G. T., Jiménez, A.-F., de Souza Rolim, G., and da Silva, R. P. (2022). Training machine learning algorithms using remote sensing and topographic indices for corn yield prediction. Remote Sensing, 14(23), 6171. https://doi.org/10.3390/rs14236171
Google Scholar	K. Kuwata et al.	Estimating Corn Yield in the United States with MODIS EVI and Machine Learning Methods	2016	Support Vector Machine, Artificial Neural Network	Kuwata, K., and Shibasaki, R. (2016). Estimating corn yield in the United States with MODIS EVI and machine learning methods. ISPRS Annals of the Photogrammetry, Remote Sensing and Spatial Information Sciences, III-8, 131–136. https://doi.org/10.5194/isprs-annals-III-8-131-2016
Google scholar	P. Fejér et al.	Predicting maize yield with a multilayer perceptron (MLP) model using multivariate field data	2025	ANN, MLP, Neural network	Fejér, P., Széles, A., Ragán, P., Juhász, C., Horváth, É., and Rátonyi, T. (2025). Predicting maize yield with a multilayer perceptron (MLP) model using multivariate field data. Precision Crop Production, 1(1), 15904. https://doi.org/10.65006/pcp.v1i01.15904
Springer	D. S. Dhaliwal et al.	Sweet corn yield prediction using machine learning models and field-level data	2023	Principal components regression, Partial least squares regression, Multiple linear regression, Random forest	Dhaliwal, D. S., and Williams, M. M. (2024). Sweet corn yield prediction using machine learning models and field-level data. Precision Agriculture, 25(1), 51–64. https://doi.org/10.1007/s11119-023-10057-1
Google Scholar	S. Khaki et al.	Simultaneous corn and soybean yield prediction from remote sensing data using deep transfer learning	2021	Random forest (RF), Deep feed forward neural network (DFNN), Regression tree (RT)	Khaki, S., Pham, H., and Wang, L. (2021). Simultaneous corn and soybean yield prediction from remote sensing data using deep transfer learning. Scientific Reports, 11, 11132. https://doi.org/10.1038/s41598-021-89779-z
Science Direct	C. Kumar et al.	Explainable machine learning models for corn yield prediction using UAV multispectral data	2025	Linear Model (GLM), K-Nearest Neighbor (KNN), Principal Component Regression (PCR), Random Forest (RF), Support Vector Machine (SVM), and Bayesian Regularized Neural Networks (BRNN).	Kumar, C., Dhillon, J., Huang, Y., and Reddy, K. (2025). Explainable machine learning models for corn yield prediction using UAV multispectral data. Computers and Electronics in Agriculture, 231, 109990. https://doi.org/10.1016/j.compag.2025.109990.
Science Direct	R. Fieuzal et al.	Estimation of corn yield using multi-temporal optical and radar satellite data and artificial neural networks	2017	Artificial neural networks	Fieuzal, R., Marais Sicre, C., and Baup, F. (2017). Estimation of corn yield using multi-temporal optical and radar satellite data and artificial neural networks. International Journal of Applied Earth Observation and Geoinformation, 57, 14–23. https://doi.org/10.1016/j.jag.2016.12.011
MDPI	P. Killeen et al.	Corn Grain Yield Prediction Using UAV-Based High Spatiotemporal Resolution Imagery, Machine Learning, and Spatial Cross-Validation	2024	Random forest (RF) and Linear regression (LR)	Killeen, P., Kiringa, I., Yeap, T., and Branco, P. (2024). Corn grain yield prediction using UAV-based high spatiotemporal resolution imagery, machine learning, and spatial cross-validation. Remote Sensing, 16(4), 683. https://doi.org/10.3390/rs16040683
MDPI	C. Kumar et al.	Multi-Stage Corn Yield Prediction Using High-Resolution UAV Multispectral Data and Machine Learning Models	2023	Linear Regression (LR), k-Nearest Neighbour (KNN), Random Forest (RF), Support Vector Regression (SVR), and Deep Neural Network (DNN)	Kumar, C., Mubvumba, P., Huang, Y., Dhillon, J., and Reddy, K. (2023). Multi-stage corn yield prediction using high-resolution UAV multispectral data and machine learning models. Agronomy, 13(5), 1277. https://doi.org/10.3390/agronomy13051277
MDPI	L. Miao et al.	Predicting China’s Maize Yield Using Multi-Source Datasets and Machine Learning Algorithms	2024	Random forest, support vector, extreme gradient boosting, BP neural network, long short-term memory network, and K-nearest neighbour regression	Miao, L., Zou, Y., Cui, X., Kattel, G. R., Shang, Y., and Zhu, J. (2024). Predicting China’s maize yield using multi-source datasets and machine learning algorithms. Remote Sensing, 16(13), 2417. https://doi.org/10.3390/rs16132417
MDPI	E. Harsányi et al.	Data Mining and Machine Learning Algorithms for Optimizing Maize Yield Forecasting in Central Europe	2023	Bagging (BG), Decision Table (DT), Random Forest (RF) and Artificial Neural Network-Multi Layer Perceptron (ANN-MLP)	Harsányi, E., Bashir, B., Arshad, S., Ocwa, A., Vad, A., Alsalman, A., Bácskai, I., Rátonyi, T., Hijazi, O., Széles, A., et al. (2023). Data mining and machine learning algorithms for optimizing maize yield forecasting in Central Europe. Agronomy, 13(5), 1297. https://doi.org/10.3390/agronomy13051297
MDPI	Y. Ren et al.	Analysis of Corn Yield Prediction Potential at Various Growth Phases Using a Process-Based Model and Deep Learning	2023	Random Forest (RF)	Ren, Y., Li, Q., Du, X., Zhang, Y., Wang, H., Shi, G., and Wei, M. (2023). Analysis of corn yield prediction potential at various growth phases using a process-based model and deep learning. Plants, 12(3), 446. https://doi.org/10.3390/plants12030446
MDPI	M. Maitah et al.	Assessment and Prediction of Maize Production Considering Climate Change by Extreme Learning Machine in Czechia	2021	Extreme learning machine (ELM)	Maitah, M., Malec, K., Ge, Y., Gebeltová, Z., Smutka, L., Blažek, V., Pánková, L., Maitah, K., and Mach, J. (2021). Assessment and Prediction of Maize Production Considering Climate Change by Extreme Learning Machine in Czechia. Agronomy, 11(11), 2344. https://doi.org/10.3390/agronomy11112344
MDPI	L. Zhang et al.	Combining Optical, Fluorescence, Thermal Satellite, and Environmental Data to Predict County-Level Maize Yield in China Using Machine Learning Approaches	2020	RF and XGBoost	Zhang, L., Zhang, Z., Luo, Y., Cao, J., and Tao, F. (2020). Combining Optical, Fluorescence, Thermal Satellite, and Environmental Data to Predict County-Level Maize Yield in China Using Machine Learning Approaches. Remote Sensing, 12(1), 21. https://doi.org/10.3390/rs12010021
MDPI	L. Su et al.	Growth Indexes and Yield Prediction of Summer Maize in China Based on Supervised Machine Learning Method	2022	Gaussian Process Regression Model, Support Vector Machine (SVM)	Su, L., Wen, T., Tao, W., Deng, M., Yuan, S., Zeng, S., and Wang, Q. (2023). Growth Indexes and Yield Prediction of Summer Maize in China Based on Supervised Machine Learning Method. Agronomy, 13(1), 132. https://doi.org/10.3390/agronomy13010132
Science Direct	G. Shao et al.	Prediction of maize crop coefficient from UAV multi sensor remote sensing using machine learning methods	2023	Linear regression-LR, polynomial regression-PR, exponential regression-ER, random forest regression-RFR, support vector regression-SVR, and deep neural network-(DNN)	Shao, G., Han, W., Zhang, H., Zhang, L., Wang, Y., and Zhang, Y. (2023). Prediction of maize crop coefficient from UAV multisensor remote sensing using machine learning methods. Agricultural Water Management, 276, 108064. https://doi.org/10.1016/j.agwat.2022.108064
Science Direct	X. Li et al.	Improving maize yield prediction at the county level from 2002 to 2015 in China using a novel deep learning approach	2022	Artificial neural networks	Li, X., Geng, H., Zhang, L., Peng, S., Xin, Q., Huang, J., Li, X., Liu, S., and Wang, Y. (2022). Improving maize yield prediction at the county level from 2002 to 2015 in China using a novel deep learning approach. Computers and Electronics in Agriculture, 202, 107356. https://doi.org/10.1016/j.compag.2022.107356
Science Direct	B. R. Sapkota et al.	Machine learning algorithms for maize yield prediction with multispectral imagery: Assessing robustness across varied growing environments	2025	Random Forest (RF), Extra Trees Regressor (ETR), K-Nearest Neighbors (KNN), Support Vector Regressor (SVR), and Linear Regression (LR)	Sapkota, B. R., Baath, G. S., Flynn, K. C., Adhikari, K., Hajda, C., and Smith, D. R. (2025). Machine learning algorithms for maize yield prediction with multispectral imagery: Assessing robustness across varied growing environments. Science of Remote Sensing, 12, 100267. https://doi.org/10.1016/j.srs.2025.100267
Science Direct	Y. Lyu et al.	Machine learning techniques and interpretability for maize yield estimation using Time-Series images of MODIS and Multi-Source data	2024	LightGBM	Lyu, Y., Wang, P., Bai, X., Li, X., Ye, X., Hu, Y., and Zhang, J. (2024). Machine learning techniques and interpretability for maize yield estimation using time-series images of MODIS and multi-source data. Computers and Electronics in Agriculture, 222, 109063. https://doi.org/10.1016/j.compag.2024.109063
Science Direct	J. Li et al.	Predicting maize yield in Northeast China by a hybrid approach combining biophysical modelling and machine learning	2023	Random Forest (RF)	Li, J., Li, G and Wang, E. (2023). Predicting maize yield in Northeast China by a hybrid approach combining biophysical modelling and machine learning. Field Crops Research, 302, 109102. https://doi.org/10.1016/j.fcr.2023.109102
Science Direct	Y. Han et al.	Prediction of maize cultivar yield based on machine learning algorithms for precise promotion and planting	2024	Random forest (RF), Levenberg–Marquardt neural network, and multilayer perceptron neural network	Han, Y., Wang, K., Yang, F., Pan, S., Liu, Z., Zhang, Q., and Zhang, Q. (2024). Prediction of maize cultivar yield based on machine learning algorithms for precise promotion and planting. Agricultural and Forest Meteorology, 355, 110123. https://doi.org/10.1016/j.agrformet.2024.110123
Science Direct	J. Desloires et al.	Out-of-year corn yield prediction at field-scale using Sentinel-2 satellite imagery and machine learning methods	2023	Random Forest (RF), Support Vector Regression (SVR), Extreme Gradient Boosting (XGBoost)	Desloires, J., Ienco, D., and Botrel, A. (2023). Out-of-year corn yield prediction at field-scale using Sentinel-2 satellite imagery and machine learning methods. Computers and Electronics in Agriculture, 209, 107807. https://doi.org/10.1016/j.compag.2023.107807
Science Direct	Q. Zhang et al.	Maize yield prediction using federated random forest	2023	Random forest	Zhang, Q., Zhao, X., Han, H., Yang, F., Pan, S., Liu, Z., Wang, K., and Zhao, C. (2023). Maize yield prediction using federated random forest. Computers and Electronics in Agriculture, 210, 107930. https://doi.org/10.1016/j.compag.2023.107930.
Research4life	S. P. G. Tahi et al.	An Experimental Analysis of Traditional Machine Learning Algorithms for Maize Yield Prediction	2024	Random Forest (RF), gradient boosting regressions (GBR), light gradient-boosting machines (light GBM), extremely randomized trees (ERT),	Tahi, S., Hounmenou, C., Houndji, V., and Glele Kakaï, R. L. (2024). An experimental analysis of traditional machine learning algorithms for maize yield prediction. Contemporary Mathematics, 6208–6224. https://doi.org/10.37256/cm.5420244481
MDPI	X. Chen et al.	Prediction of Maize Yield at the City Level in China Using Multi-Source Data	2021	Random forest (RF), extreme gradient boosting (Xgboost), and support vector machine (SVM)	Chen, X., Feng, L., Yao, R., Wu, X., Sun, J., and Gong, W. (2021). Prediction of maize yield at the city level in China using multi-source data. Remote Sensing, 13(1), 146. https://doi.org/10.3390/rs13010146
Google Scholar	K. Matsumura et al	Maize yield forecasting by linear regression and artificial neural networks in Jilin, China	2014	Linear regression and Artificial neural networks	Matsuura, K., Gaitan, C., Hsieh, W., and Cannon, A. (2014). Maize yield forecasting by linear regression and artificial neural networks in Jilin, China. The Journal of Agricultural Science, 152(6), 107–118. https://doi.org/10.1017/S0021859614000392
MDPI	D. Shamsuddin et al.	Multimodal Deep Learning Integration of Image, Weather, and Phenotypic Data Under Temporal Effects for Early Prediction of Maize Yield	2024	Deep neural network	Shamsuddin, D., Danilevicz, M. F., Al-Mamun, H. A., Bennamoun, M., and Edwards, D. (2024). Multimodal deep learning integration of image, weather, and phenotypic data under temporal effects for early prediction of maize yield. Remote Sensing, 16(21), 4043. https://doi.org/10.3390/rs16214043
MDPI	A. Attia et al.	Coupling Process-Based Models and Machine Learning Algorithms for Predicting Yield and Evapotranspiration of Maize in Arid Environments	2022	Linear regression, ridge regression, lasso regression, K-nearest neighbors, random forest, and XGBoost	Attia, A., Govind, A., Qureshi, A. S., Feike, T., Rizk, M. S., Shabana, M. M. A., and Kheir, A. M. S. (2022). Coupling process-based models and machine learning algorithms for predicting yield and evapotranspiration of maize in arid environments. Water, 14(22), 3647. https://doi.org/10.3390/w14223647
MDPI	W. Zhou et al.	A Prediction Model of Maize Field Yield Based on the Fusion of Multitemporal and Multimodal UAV Data: A Case Study in Northeast China	2023	Convulational Neural Networks	Zhou, W., Song, C., Liu, C., Fu, Q., An, T., Wang, Y., Sun, X., Wen, N., Tang, H., and Wang, Q. (2023). A prediction model of maize field yield based on the fusion of multitemporal and multimodal UAV data: A case study in Northeast China. Remote Sensing, 15(14), 3483. https://doi.org/10.3390/rs15143483
Google Scholar	O. Ennaji et al.	Gradient boosting for yield prediction of elite maize hybrid ZhengDan 958	2024	eXtreme Gradient Boost (XGB), Gradient Boosting (GBR), Random Forest (RF), Artificial Neural Network (ANN), Support Vector Regression (SVR), Linear Regression (LR), Decision Trees (DT), and K-Nearest Neighbors (KNN).	Ennaji, O., Baha, S., Vergutz, L., and El Allali, A. (2024). Gradient boosting for yield prediction of elite maize hybrid ZhengDan 958. PLOS ONE, 19(6), e0315493. https://doi.org/10.1371/journal.pone.0315493
Research4life	Ge et al.	Enhancing yield prediction in maize breeding using UAV-derived RGB imagery: a novel classification-integrated regression approach	2025	Support Vector Machine (SVM), Decision Tree (DT), and Random Forest (RF)	Ge H, Zhang Q, Shen M, Qin Y, Wang L and Yuan C (2025) Enhancing yield prediction in maize breeding using UAV-derived RGB imagery: a novel classification-integrated regression approach. Front. Plant Sci. 16:1511871. doi:10.3389/fpls.2025.1511871
MDPI	M. F. Danilevicz et al.	Maize Yield Prediction at an Early Developmental Stage Using Multispectral Images and Genotype Data for Preliminary Hybrid Selection	2021	Convolutional neural networks (CNN), Random forest and XGBoost	Danilevicz, M. F., Bayer, P. E., Boussaid, F., Bennamoun, M., and Edwards, D. (2021). Maize Yield Prediction at an Early Developmental Stage Using Multispectral Images and Genotype Data for Preliminary Hybrid Selection. Remote Sensing, 13(19), 3976. https://doi.org/10.3390/rs13193976
Science Direct	C. Lu et al.	In-season maize yield prediction in Northeast China: The phase-dependent benefits of assimilating climate forecast and satellite observations	2024	Random Forest	Lu, C., Leng, G., Liao, X., Tu, H., Qiu, J., Li, J., Huang, S., and Peng, J. (2024). In-season maize yield prediction in Northeast China: The phase-dependent benefits of assimilating climate forecast and satellite observations. Agricultural and Forest Meteorology, 358, 110242. https://doi.org/10.1016/j.agrformet.2024.110242
Science Direct	S. Sunoj et al.	Maize grain and silage yield prediction of commercial fields using high-resolution UAS imagery	2022	Random Forest, Support Vector Machine	Sunoj, S., Yeh, B., Marcaida, M. III, Longchamps, L., van Aardt, J., and Ketterings, Q. M. (2023). Maize grain and silage yield prediction of commercial fields using high-resolution UAS imagery. Biosystems Engineering, 235, 137–149. https://doi.org/10.1016/j.biosystemseng.2023.09.010
Science Direct	Y. Zhou et al.	Enhancing corn yield prediction: Optimizing data quality or model complexity?	2024	Random Forest and Gradient Boosting	Zhou, Y., Ma, S., Zhang, H., and Aakur, S. (2024). Enhancing corn yield prediction: Optimizing data quality or model complexity? Smart Agricultural Technology, 9, 100671.
Science Direct	Y. Guo et al.	Predicting grain yield of maize using a new multispectral-based canopy volumetric vegetation index	2024	Backpropagation neural network (BP) and random forest (RF)	Guo, Y., Fu, Y. H., Chen, S., Hao, F., Zhang, X., de Beurs, K., and He, Y. (2024). Predicting grain yield of maize using a new multispectral-based canopy volumetric vegetation index. Ecological Indicators, 166, 112295. https://doi.org/10.1016/j.ecolind.2024.112295
Google scholar	Azrai et al	Optimizing ensembles machine learning, genetic algorithms, and multivariate modeling for enhanced prediction of maize yield and stress tolerance index	2024	SVM, KNN, and RF	Azrai, M., Aqil, M., Andayani, N. N., Efendi, R., Suarni, S., Suwardi, J., Jihad, M., Zainuddin, B., Salim, B., Muliadi, A., Yasin, M., Hannan, M. F. I., Rahman, and Syam, A. (2024). Optimizing ensembles machine learning, genetic algorithms, and multivariate modeling for enhanced prediction of maize yield and stress tolerance index. Frontiers in Sustainable Food Systems, 8, 1334421.
Springer	I. K. Fernandes et al.	Using machine learning to combine genetic and environmental data for maize grain yield predictions across multi-environment trials	2024	LightGBM, Convolutional neural networks (CNN), Random Forest	Fernandes, I. K., Vieira, C. C., Dias, K. O. G., et al. (2024). Using machine learning to combine genetic and environmental data for maize grain yield predictions across multi-environment trials. Theoretical and Applied Genetics, 137, 189. https://doi.org/10.1007/s00122-024-04687-w
MDPI	L. Meng et al.	Predicting Maize Yield at the Plot Scale of Different Fertilizer Systems by Multi-Source Data and Machine Learning Methods	2021	Linear regression (LR), K-nearest neighbor (KNN), support vector machines (SVM), Gaussian process regression (GPR), adaptive boost (AB), and random forests (RF).	Meng, L., Liu, H., L. Ustin, S., and Zhang, X. (2021). Predicting Maize Yield at the Plot Scale of Different Fertilizer Systems by Multi-Source Data and Machine Learning Methods. Remote Sensing, 13(18), 3760. https://doi.org/10.3390/rs13183760

Several studies evaluated multiple machine learning models for benchmarking purposes. Table 5 reports all models assessed in each study. Where explicitly stated by the original authors, the best-performing model was identified based on the primary evaluation metric (e.g., RMSE or *R*^2^).

### 3.3 Machine learning algorithms used

Addressing RQ1, analysis of the algorithms used (Table 6) indicates that tree-based bagging methods dominate agricultural yield prediction studies. Because many studies evaluated more than one machine learning algorithm for benchmarking purposes, the total frequency of algorithms reported in Table 6 exceeds the number of reviewed studies. Random Forest was the most frequently applied algorithm, appearing in 40 of 81 studies (49.4 percent), followed by the Extremely Randomized Tree in three studies. Boosting algorithms ranked second, with XGBoost used in 13 studies (16.1 percent) and LightGBM in five, demonstrating a growing preference for gradient-boosted ensemble models that enhance predictive accuracy and robustness. Support Vector Machine approaches, including Support Vector Machine (10 studies) and Support Vector Regression (9 studies), accounted for 23.5 percent, reflecting their sustained relevance for high-dimensional and nonlinear datasets. Neural and deep learning models also featured prominently, with Artificial Neural Networks (9 studies), Convolutional Neural Networks (4), Deep Neural Networks (4), and Back Propagation Networks (2) showing increasing adoption in data-rich agricultural contexts. Linear models such as Lasso, Ridge, and Multiple Linear Regression collectively appeared in 12 studies, primarily as benchmark or interpretable baselines. Instance-based learning methods, represented by K-Nearest Neighbor (9), and Gaussian Process Regression (3) were used in specific applications emphasizing spatial proximity and uncertainty estimation.

**Table 6:** Frequency of machine learning algorithms used across the reviewed studies.

Category	Machine learning algorithms	# of times used
Bagging algorithms	Random Forest	40
	Extremely randomized tree	3
Boosting algorithms	Extreme gradient boosting (XGBoost)	13
	LightGBM Models	5
Support Vector Machines	Support Vector Machine	10
	Support Vector Regression	9
Neural Network and Deep Learning	Artificial Neural Network	9
	Convolutional Neural Networks	4
	Back propagation Neural Network	2
	Deep Neural Network	4
Linear Models	Lasso Regression	5
	Ridge Regression	4
	Multiple Linear Regression	3
Instance based Learning	K-Nearest Neighbor	9
Gaussian Process Models	Gaussian Process Regression	3

Note that several studies implemented multiple algorithms for comparison; therefore, total frequencies exceed the number of included studies (*n* = 81).

### 3.4 Features used in model development

In response to RQ2, the review identified consistent reliance on four major feature categories: environmental variables (50 uses), remote sensing data (45), soil characteristics (22), and crop yield data (21) (Table 7). Environmental variables such as rainfall and temperature were the most frequently used predictors (e.g., Guo et al., 2023; Muthoni et al., 2021), while remote sensing inputs including NDVI and EVI derived from UAV and satellite imagery were widely applied for spatial yield estimation (Bolton and Friedl, 2013; Cheng et al., 2022; Zhang et al., 2025). Soil characteristics were incorporated in fewer studies, mainly to capture site-specific variability (Asamoah et al., 2024; Li et al., 2022). The growing integration of multi-source and multi-temporal datasets indicates a transition toward holistic data fusion strategies that combine climatic, soil, and remote-sensing information within unified modeling frameworks. Evidence from recent hybrid modeling studies shows that integrating process-based crop models with machine learning and Earth-observation data enhances ecological realism while maintaining high predictive accuracy, particularly for yield gap and productivity assessments (Kheir et al., 2025a; van Klompenburg et al., 2020).

**Table 7:** Input features used in the reviewed studies.

Feature	# of times used
Crop yield data	21
Soil data	22
Environmental data	50
Remote sensing data	45
Maize trials data	1
Questionnaires	2
Agronomic data	2
Geographic data	1
Elevation data	1
Cropland data	4
Phenotypic data	2
Genetic data	1
Crop data	1

Note that individual studies often used multiple data sources; therefore, total counts exceed the number of reviewed studies (*n* = 81).

### 3.5 Model evaluation parameters

Model evaluation within the reviewed literature is dominated by Root Mean Square Error (RMSE) and Coefficient of Determination (*R*^2^), used in 51 and 46 studies, respectively, (Table 8). These metrics were commonly reported in studies using satellite, UAV, and multi-source inputs (e.g., Bolton and Friedl, 2013; Cheng et al., 2022; Guo et al., 2023; Asamoah et al., 2024). MAE and MAPE were used less frequently as complementary indicators of absolute and percentage error. This pattern suggests partial convergence around a core set of deterministic regression metrics, but reporting remains heterogeneous because relatively few studies explicitly report uncertainty measures, baseline skill comparisons, or validation designs in a standardized way. Accordingly, the field has not yet reached full methodological standardization, even though RMSE and *R*^2^ remain the most common evaluation measures.

**Table 8:** All evaluation parameters used.

Key	Evaluation parameter	# of times used
RMSE	Root Mean Square Error	51
*R*^2^	Coefficient of Determination	46
MAE	Mean Absolute Error	17
MAPE	Mean Absolute Percentage Error	7
	Accuracy	4
MARE	Mean Absolute Relative Error	2
RAE	Relative Absolute Error	2
NRMSE	Normalized Root Mean Square Error	2
	Pearson Correlation Coefficient	2
MSE	Mean Square Error	1
	Percentage bias	1

### 3.6 Challenges in the application of machine learning for maize yield prediction

**Figure 5:**
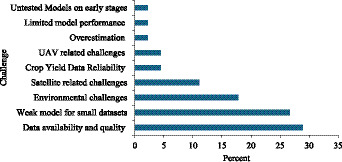
Challenges in application of machine learning for maize yield prediction.

Despite substantial progress, 45 of the 81 studies acknowledged limitations constraining ML model scalability and transferability (Figure 5). Data availability and data quality were the most frequently reported challenges, especially in studies relying on smallholder field observations, limited ground-truth samples, or short time series (Li et al., 2022; Muthoni et al., 2021; Asamoah et al., 2024). Remote-sensing-based studies also reported operational problems linked to cloud contamination, revisit gaps, mixed pixels, and inconsistencies between image timing and crop phenology, which can reduce predictive stability at field and regional scales (Bolton and Friedl, 2013; Kenduiywo and Miller, 2024). In UAV-based studies, weather sensitivity, mission logistics, and restricted spatial coverage can limit repeatability and scalability, even when local prediction accuracy is strong (Guo et al., 2023; de Villiers et al., 2024). A further recurring constraint is the lack of harmonized ground-truth data for robust validation across locations and seasons, which weakens model transferability and cross-study comparability (Gholami et al., 2022; van Klompenburg et al., 2020). Collectively, these findings underscore the need for better data governance, standardized validation workflows, and more interpretable modeling pipelines.

## 4 Discussion

The findings of this systematic review highlight the accelerating application of machine learning (ML) in maize yield prediction, with a marked increase in publications after 2020 reflecting a global shift toward data-driven agriculture. Similar to the observations of van Klompenburg et al. (2020), this trend shows the growing recognition of ML as a critical decision-support tool for yield forecasting and food-security planning. The reviewed studies demonstrate that ML algorithms, particularly Random Forest (RF), Gradient Boosting Decision Trees (GBDT), and Artificial Neural Networks (ANN), have become dominant approaches for maize yield modeling owing to their robustness in handling non-linear, high-dimensional datasets (Guo et al., 2023; Olayinka et al., 2025; Saha et al., 2025). These results agree with the global pattern noted in previous systematic reviews, where neural networks and tree-based ensembles outperformed classical regression techniques (van Klompenburg et al., 2020; Dwivedi et al., 2019).

### 4.1 Algorithmic performance and feature importance

Across the 81 studies analyzed, Random Forest emerged as the most frequently applied algorithm, followed by Gradient Boosting and ANN. The popularity of RF lies in its capacity to minimize overfitting and manage multicollinearity among predictors, while GBDT’s iterative learning enables high predictive precision from limited datasets (Chigwada et al., 2023; Asamoah et al., 2024). ANN-based models, including hybrid architectures such as CNN-LSTM, demonstrated superior performance when integrating spatiotemporal variables like vegetation indices and climatic parameters (Taremwa et al., 2025; Cheng et al., 2022). Consistent with global findings by Zhang et al. (2025) and Hamim et al. (2025), these hybrid and deep-learning frameworks outperform shallow models, particularly when high-resolution remote-sensing data are incorporated. However, interpretability remains a concern confirming the earlier critiques that “black-box” ML models limit agronomic insight and policy uptake (van Klompenburg et al., 2020; Muriuki et al., 2023). Reported performance advantages of Random Forest, XGBoost, and deep learning models often coincide with richer datasets, longer time series, or spatially overlapping training and testing data. As such, observed differences cannot be attributed solely to algorithmic superiority. When evaluated under comparable data and validation conditions, performance gaps among algorithm classes are frequently reduced, underscoring the importance of experimental design in model assessment.

### 4.2 Input variables and data integration

Input features can be broadly categorized into static variables such as soil properties and topography, and dynamic variables such as weather conditions and vegetation indices. Pre-season predictors are more relevant for early warning and planning, whereas in-season indicators, particularly time-series vegetation indices, tend to drive finer-scale yield estimation accuracy (Bolton and Friedl, 2013; Cheng et al., 2022; Zhang et al., 2025). This review confirms that environmental and biophysical variables, such as temperature, rainfall, soil type, and vegetation indices, are the most frequently used inputs for maize yield modeling. The increasing use of UAV-based hyperspectral imagery and Sentinel-2 satellite data signifies a methodological transition toward high-spatial-resolution datasets, especially in African and Asian contexts (de Villiers et al., 2024; Kenduiywo and Miller, 2024). Integrating climatic, soil, and remote-sensing data has proven crucial for improving yield prediction accuracy, as observed in studies from China, Ghana, and Malawi (Guo et al., 2023; Muthoni et al., 2021; Asamoah et al., 2024; Li et al., 2022). These patterns align with van Klompenburg et al.’s (2020) conclusion that multi-source data fusion substantially enhances model generalization.

### 4.3 Geographical and methodological gaps

The review reveals pronounced geographical disparities in the distribution of research on machine-learning-based maize yield prediction. Asia and the Americas account for more than 65 percent of the reviewed studies, whereas African contributions remain underrepresented at approximately 23 percent. This imbalance is consistent with earlier reviews and reflects persistent inequalities in digital infrastructure, data availability, and research investment across regions (van Klompenburg et al., 2020; Liakos et al., 2018). Studies from countries such as Malawi, Ghana, and South Africa frequently rely on publicly available satellite products or relatively small field experiments, which can constrain model calibration, external validation, and scalability (Adisa et al., 2019; Li et al., 2022; Asamoah et al., 2024; Kenduiywo and Miller, 2024). The limited availability of open and harmonized agricultural datasets further hampers reproducibility and cross-regional comparability of model outputs, reinforcing calls for national data repositories and collaborative research platforms (Gholami et al., 2022). In addition, explicit varietal information was uncommon among the reviewed studies, suggesting that genotype-specific responses remain underrepresented in current maize-yield models and may limit transferability across cultivars and agroecological zones.

### 4.4 Evaluation metrics and benchmarking

Most studies evaluated model performance using standard regression metrics including *R*^2^, RMSE, MAE, and MAPE, but few used a common benchmarking framework. This mirrors van Klompenburg et al.’s (2020) observation that inconsistent evaluation practices hinder cross-study comparison. Advanced ensemble and deep-learning models generally achieved high predictive accuracy in data-rich contexts, yet performance often declined in smallholder systems with sparse or heterogeneous training data (Guo et al., 2023; Asamoah et al., 2024; Taremwa et al., 2025). Recent studies also show that validation design materially affects apparent performance: spatially structured approaches such as spatial cross-validation can produce substantially more conservative and realistic estimates than random k-fold validation when spatial autocorrelation is present (Killeen et al., 2024). Metric choice should therefore be guided by analytical purpose rather than popularity. RMSE is useful when large errors carry disproportionate cost (Chai and Draxler, 2014), MAE offers a more robust measure of average absolute error magnitude error than RMSE (Willmott and Matsuura, 2005), and MAPE is only appropriate when observed yields are strictly positive and percentage error is substantively meaningful (Hyndman and Koehler, 2006). Median-based measures such as MedAE or MedAPE can also be informative when error distributions are skewed (Hewamalage et al., 2022). *R*^2^ should be interpreted as a supplementary goodness-of-fit indicator rather than a stand-alone basis for model selection, and agreement statistics should be chosen cautiously because widely used indices can be misleading when they do not match the decision problem (Sapra, 2014; Harrell, 2015; Willmott and Matsuura, 2005). The near absence of probabilistic metrics, uncertainty intervals, and skill scores relative to baseline models remains a major gap.

### 4.5 Implications for developing *de novo* maize yield prediction models

Based on the reviewed literature, a minimal yet robust feature set for developing a *de novo* maize yield prediction model should include: (i) key environmental variables such as cumulative rainfall and temperature during critical growth stages; (ii) remotely sensed vegetation indices, particularly NDVI or EVI derived from satellite or UAV imagery; and (iii) basic soil properties such as soil texture or organic matter where available. This combination represents the most frequently and consistently used predictors across studies and balances predictive performance with data availability (Bolton and Friedl, 2013; Muthoni et al., 2021; Guo et al., 2023). Model evaluation should rely on a complementary metric set rather than on a single dominant indicator. A practical combination is RMSE to reflect the penalty of large errors, MAE (or MedAE where outliers are influential) to represent typical absolute error, and *R*^2^ as a supplementary measure of explained variance. MAPE or MedAPE may be added only when all observed yields are positive and percentage interpretation is meaningful. Where spatial data are used, these metrics should be reported under spatially or temporally appropriate validation designs to improve robustness and comparability (van Klompenburg et al., 2020; Killeen et al., 2024; Pontius and Millones, 2011).

### 4.6 Challenges and opportunities

Despite remarkable progress, challenges persist. Data scarcity, poor ground-truth quality, and limited computational resources remain barriers to widespread ML adoption in low-income countries (Muriuki et al., 2023; Omokpariola et al., 2025). Furthermore, several studies incorporate biophysical predictors but omit socio-economic and management variables such as fertilizer application, irrigation, planting density, and farmer practices, even though these factors materially influence yield outcomes (Anghileri et al., 2024; Faloye et al., 2025; Asamoah et al., 2024). The reviewed evidence also suggests that interpretability tools are still not routinely integrated into maize-yield models, which may limit trust and operational uptake. Addressing these gaps will enhance the interpretability, trust, and scalability of ML-based decision-support systems for agrifood resilience.

### 4.7 Analytical framework for algorithm performance across data and scale conditions

Beyond reporting algorithm frequencies, the reviewed literature reveals systematic patterns linking algorithm performance to data availability, spatial scale, and application context. Tree-based ensemble models such as Random Forest and Gradient Boosting dominate maize yield prediction primarily because they perform robustly under moderate data volumes, handle multicollinearity effectively, and require limited parameter tuning (Chigwada et al., 2023; Asamoah et al., 2024; Guo et al., 2023). These characteristics make them particularly suitable for regional and national-scale applications and for data-constrained smallholder systems (Li et al., 2022; Kenduiywo and Miller, 2024). In contrast, deep-learning models tend to perform best in data-rich environments with long time series or high-resolution imagery, such as commercial farming systems or experimental settings, but they can underperform or overfit when applied to sparse or heterogeneous datasets (Taremwa et al., 2025; Zhang et al., 2025). Simpler regression-based models remain competitive in smaller datasets or shorter time-series contexts, especially where interpretability is prioritized (Bolton and Friedl, 2013). This synthesis highlights that algorithm dominance reflects data and scale suitability rather than intrinsic superiority.

### 4.8 Implications for developing countries

For countries such as Malawi, where maize supports both food and income security, the adoption of machine-learning-based yield prediction systems offers substantial transformative potential. Previous studies demonstrate that accurate and timely yield forecasts can enhance policy responsiveness, resource allocation, and risk management in climate-sensitive agricultural systems (Chitsiko et al., 2022; Nyirenda et al., 2021; Gholami et al., 2022). Recent advances in geospatial integration and cloud-enabled analytics further improve the feasibility of scalable early-warning systems, but their deployment depends on complementary investments in digital infrastructure, human capacity, and robust data governance (Anghileri et al., 2024; Kenduiywo and Miller, 2024). Hybrid process-based and machine-learning frameworks also offer a promising pathway for operational decision support because they combine biophysical realism with predictive flexibility (Kheir et al., 2025a,b,c). Within this context, the present review contributes by synthesizing evidence on algorithms, predictors, evaluation practices, and implementation gaps that are most relevant to maize-dependent developing countries.

## 5 Conclusion

This systematic review synthesizes evidence from 81 studies applying machine learning approaches to maize yield prediction and demonstrates the growing importance of data driven methods in contemporary agricultural research. The findings show that ensemble and neural network based algorithms, particularly Random Forest, Gradient Boosting, and Artificial Neural Networks, dominate the literature and consistently deliver superior predictive performance relative to traditional statistical models. Their effectiveness stems from an enhanced ability to capture complex, non linear interactions among climatic, environmental, and biophysical variables, thereby improving the accuracy and scalability of yield forecasts across diverse production systems (van Klompenburg et al., 2020; Guo et al., 2023; Olayinka et al., 2025). Since 2021, research activity has expanded rapidly, reflecting advances in remote sensing technologies, increased data availability, and improved computational capacity. Nevertheless, this growth remains geographically uneven. Asia and the Americas account for the majority of published studies, while Africa contributes less than a quarter, despite maize being a cornerstone of food security across the continent. This imbalance highlights persistent disparities in data infrastructure, research capacity, and investment, particularly in sub Saharan Africa (Adisa et al., 2019; Muriuki et al., 2023).

Although machine learning models demonstrate high predictive accuracy in data rich and controlled environments, several limitations constrain their broader applicability. Challenges related to data quality, model interpretability, and transferability across agro ecological contexts remain widespread. Most existing models rely predominantly on biophysical inputs and are often calibrated using publicly available satellite products or small scale field experiments, which limits their generalizability. In addition, the reviewed literature reveals limited consideration of genetic heterogeneity, as yield predictions are commonly generated without explicit differentiation among local landraces, improved hybrids, or genetically modified maize varieties. Such genetic differences can influence crop responses to environmental and management conditions, with important implications for model robustness and cross regional transferability. Addressing these methodological gaps is essential for advancing the reliability and policy relevance of machine learning based yield prediction systems.

A major finding of this review is the limited integration of socio economic and management variables within existing modeling frameworks. Factors such as access to fertilizer, labor availability, land tenure security, extension services, and policy support play a central role in shaping farmer decision making and production outcomes, especially in smallholder dominated systems. While some studies emphasize the performance of machine learning models under biophysical data constraints, broader food security and yield forecasting research underscores the importance of incorporating socio economic and management indicators to improve contextual relevance and practical applicability (Chitsiko et al., 2022; Gholami et al., 2022; Omokpariola et al., 2025). Integrating these dimensions offers a pathway to capturing the human and institutional drivers of yield variability that directly influences harvested output in terms of tons or kilograms, thereby strengthening the explanatory and predictive power of future models.

A major finding of this review is the limited integration of socio-economic and management variables within existing modeling frameworks. Factors such as fertilizer access, irrigation, extension support, and household decision-making can materially shape yield outcomes, especially in smallholder systems. Yet most reviewed models rely predominantly on biophysical inputs, even though studies that combine agronomic management, household survey, and remote-sensing information demonstrate the added contextual value of such integration (Anghileri et al., 2024; Gholami et al., 2022; Faloye et al., 2025). Integrating these dimensions offers a pathway to capturing the human and institutional drivers of yield variability, thereby strengthening the explanatory and predictive power of future models.

Overall, this review concludes that machine learning holds substantial promise for enhancing the precision, timeliness, and scalability of maize yield prediction. Realizing this potential, however, will depend on embedding machine learning innovations within inclusive and transparent data ecosystems, national agricultural strategies, and sustained capacity building efforts. Progress in this field requires coordinated action across research, policy, and practice. Priority areas include the development of open and interoperable agricultural data systems that integrate climatic, soil, and management information, the adoption of hybrid and explainable modeling frameworks that improve interpretability and trust, and targeted investments in data science and computational agronomy training, particularly in low- and middle-income countries. Equally important is the integration of socio economic and policy variables to ensure that model outputs reflect the realities of farmer decision making and support equitable, climate resilient food systems. Through such integrated and context sensitive approaches, machine learning can evolve from a promising analytical tool into a transformative component of sustainable agrifood decision making. Further, most reviewed studies frame yield prediction as a static modeling task, with limited attention to uncertainty quantification, non-stationarity, or decision-relevant forecasting horizons. Operational deployment requires explicit handling of uncertainty, probabilistic outputs, and robustness to changing climate and management conditions, which remain underexplored in the current literature.

## Data Availability

The original contributions presented in the study are included in the article/supplementary material, further inquiries can be directed to the corresponding author/s.

## Appendix

Studies that passed all exclusion criteria available at https://drive.google.com/drive/folders/1Ny_yGPCXNqZLGM-bS2TurQJbBICcfUu0?usp=sharing.

## References

[ref1] Adisa O. M. Mutanga O. Botai J. O. (2019). Application of artificial neural networks for predicting maize production in South Africa. Sustainability 11:5343. doi: 10.3390/su11195343

[ref2] Anghileri D. Chibarabada T. P. Gadedjisso-Tossou A. Craig A. Li C. Lu Y. . (2024). Understanding the maize yield gap in southern Malawi by integrating ground and remote-sensing data, models, and household surveys. Agric. Syst. 218:103962. doi: 10.1016/j.agsy.2024.103962

[ref3] Asamoah E. Gyasi-Agyei Y. Adu-Appiah S. (2024). Random-forest machine learning for maize yield and agronomic efficiency prediction in Ghana. Agric. Water Manag. 292:108769. doi: 10.1016/j.agwat.2024.108769 PMC11403005 39286064

[ref4] Bolton D. K. Friedl M. A. (2013). Forecasting crop yield using remotely sensed vegetation indices and crop phenology metrics. Agric. For. Meteorol. 173, 74–84. doi: 10.1016/j.agrformet.2013.01.007

[ref5] Chai T. Draxler R. R. (2014). Root mean square error (RMSE) or mean absolute error (MAE)? – arguments against avoiding RMSE in the literature. Geosci. Model Dev. 7, 1247–1250. doi: 10.5194/gmd-7-1247-2014

[ref6] Cheng M. Wu Z. Zhao X. (2022). Combining multi-indicators with machine-learning algorithms for maize yield early prediction at the county level in China. Remote Sens. Environ. 276:113074. doi: 10.1016/j.rse.2022.113074

[ref7] Chigwada T. Matsika R. Chirima M. (2023). Maize crop yield prediction model using machine learning. Open Geosci. 15, 101–112. doi: 10.1515/geo-2023-0010

[ref8] Chitsiko R. J. Mutanga O. Dube T. Kutywayo D. (2022). Review of current models and approaches used for maize crop yield forecasting in sub-Saharan Africa and their potential use in early warning systems. Phys. Chem. Earth Parts A/B/C 127:103199. doi: 10.1016/j.pce.2022.103199

[ref9] de Villiers C. D. Botha N. Lourens A. (2024). Assessing maize yield spatiotemporal variability using unmanned aerial vehicles and machine learning. Remote Sens. 16:276. doi: 10.3390/rs16020276

[ref10] Dwivedi Y. K. Hughes L. Ismagilova E. Aarts G. Coombs C. Crick T. . (2019). Artificial intelligence (AI): multidisciplinary perspectives on emerging challenges, opportunities, and agenda for research, practice and policy. Int. J. Inf. Manag. 57:101994. doi: 10.1016/j.ijinfomgt.2019.08.002

[ref11] Faloye O. T. Akinyemi O. P. Bamigboye G. O. (2025). Forecasting maize yield from growth parameters using machine learning in biochar-amended soils under drip irrigation. Comput. Electron. Agric. 225:109043. doi: 10.1016/j.compag.2025.109043

[ref12] Gholami S. Knippenberg E. Campbell J. Andriantsimba D. Kamle A. Parthasarathy P. . (2022). Food security analysis and forecasting: a machine learning case study in southern Malawi. Data Policy 4. doi: 10.1017/dap.2022.25

[ref13] Guo Y. Zhang Q. Li J. Han Y. (2023). Comparison of different machine learning algorithms for predicting maize grain yield using UAV-based hyperspectral images. Comput. Electron. Agric. 206:107662. doi: 10.1016/j.compag.2023.107662

[ref14] Hamim A. M. Mohaimin A. Ishmum A. Ifty R. Patwary M. (2025). Advances in machine learning for crop yield prediction: a comprehensive review of techniques, trends, and challenges. 2025 International Conference on Electrical, Computer and Communication Engineering (ECCE), 1–6. doi: 10.1109/ECCE64574.2025.11013031

[ref15] Harrell F. E. Jr. (2015). Regression Modeling Strategies: With Applications to Linear Models, Logistic and Ordinal Regression, and Survival Analysis. 2nd Edn. Cham: Springer. doi: 10.1007/978-3-319-19425-7

[ref16] Hewamalage H. Ackermann K. Bergmeir C. (2022). Forecast evaluation for data scientists: common pitfalls and best practices. Data Min. Knowl. Disc. 37, 788–832. doi: 10.1007/s10618-022-00894-5, 36504672 PMC9718476

[ref17] Hyndman R. J. Koehler A. B. (2006). Another look at measures of forecast accuracy. Int. J. Forecast. 22, 679–688. doi: 10.1016/j.ijforecast.2006.03.001

[ref18] Jabed M. A. Azmi Murad M. A. (2024). Crop yield prediction in agriculture: a comprehensive review of machine learning and deep learning approaches, with insights for future research and sustainability. Heliyon 10:e40836. doi: 10.1016/j.heliyon.2024.e40836, 39720079 PMC11667600

[ref19] Jhajharia D. Sharma N. Mathur P. (2025). A machine learning model for crop yield prediction using remote sensing data. Int. Res. J. Multidis. Scope 6, 577–590. doi: 10.47857/irjms.2025.v06i02.03182

[ref20] Kenduiywo B. K. Miller S. (2024). Seasonal maize yield forecasting in south and east African countries using hybrid earth-observation models. Agric. For. Meteorol. 340:109589. doi: 10.1016/j.agrformet.2024.109589 PMC11283090 39071562

[ref21] Kheir A. M. S. Gosme M. Bakhtiary N. Kephe P. Assou J. Langhof M. . (2025c). Towards a comprehensive decision support system for agroforestry systems. Environ. Res. Lett. 20:124016. doi: 10.1088/1748-9326/ae1cd5

[ref22] Kheir A. M. S. Shabana M. M. A. Ali M. G. M. Attia A. Abd El-Aziz M. A. Feike T. (2025a). “Fusion of process-based models, machine learning, and remote sensing for yield gap assessment,” in Resilient Agroecosystems (Sustainability Sciences in Asia and Africa), eds. Zohry A. E. H. Ouda S. (Singapore: Springer).

[ref23] Kheir A. M. S. Shabana M. M. A. Ali M. G. M. Attia A. Abd El-Aziz M. A. Feike T. (2025b). Hybridization of process-based models, remote sensing, and machine learning for enhanced spatial predictions of wheat yield and quality. Comput. Electron. Agric. 223:110317. doi: 10.1016/j.compag.2025.110317

[ref24] Killeen P. Kiringa I. Yeap T. Branco P. (2024). Corn grain yield prediction using UAV-based high spatiotemporal resolution imagery, machine learning, and spatial cross-validation. Remote Sens. 16:683. doi: 10.3390/rs16040683

[ref25] Li C. Chimimba E. G. Kambombe O. Brown L. A. Chibarabada T. P. Lu Y. . (2022). Maize yield estimation in intercropped smallholder fields using satellite data in southern Malawi. Remote Sens. 14, 1–14. doi: 10.3390/rs14102458

[ref26] Liakos K. G. Busato P. Moshou D. Pearson S. Bochtis D. (2018). Machine learning in agriculture: a review. Sensors 18:2674. doi: 10.3390/s18082674, 30110960 PMC6111295

[ref27] Muriuki J. Hudson D. Fuad S. (2023). The impact of conflict on food security: evidence from household data in Ethiopia and Malawi. Agric. Food Secur. 12:41. doi: 10.1186/s40066-023-00447-z

[ref28] Muthoni F. Nyirenda H. Mwase W. (2021). Machine-learning models accurately predict maize grain yields in conservation-agriculture systems in southern Africa. Field Crop Res. 274:108316. doi: 10.1016/j.fcr.2021.108316

[ref29] Ng’ombe A. Sithole M. Musafiri C. M. Kiboi M. Sales T. Kayira M. . (2024). Uptake determinants of climate-smart agricultural practice for greening smallholder groundnut value chain: evidence from Malawi. Cleaner Circ. Bioecon. 9:100123. doi: 10.1016/j.clcb.2024.100123

[ref30] Nyirenda H. Mwangomba W. Nyirenda E. M. (2021). Delving into possible missing links for attainment of food security in Central Malawi: farmers’ perceptions and long term dynamics in maize (*Zea mays* L.) production. Heliyon 7:e07130. doi: 10.1016/j.heliyon.2021.e07130, 34095596 PMC8167231

[ref31] Olayinka T. C. Adewumi A. O. Adebiyi A. A. (2025). A data-driven machine learning approach toward improved maize crop production. Comput. Electron. Agric. 225:109015. doi: 10.1016/j.compag.2025.109015

[ref32] Omokpariola D. O. Agbanu-Kumordzi C. Samuel T. Kiswii L. Moses G. S. Adelegan A. M. (2025). Climate change, crop yield, and food security in sub-Saharan Africa. Discover Sustain. 6:678. doi: 10.1007/s43621-025-01580-4

[ref33] Owens J. K. (2021). Systematic reviews: brief overview of methods, limitations, and resources. Nurse Author Ed. 31, 69–72. doi: 10.1111/nae2.28

[ref34] Page M. J. McKenzie J. E. Bossuyt P. M. Boutron I. Hoffmann T. C. Mulrow C. D. . (2021). The PRISMA 2020 statement: an updated guideline for reporting systematic reviews. BMJ 372:n71. doi: 10.1136/bmj.n71, 33782057 PMC8005924

[ref35] Pontius R. G. Jr. Millones M. (2011). Death to kappa: birth of quantity disagreement and allocation disagreement for accuracy assessment. Int. J. Remote Sens. 32, 4407–4429. doi: 10.1080/01431161.2011.552923

[ref36] Saha S. Kucher O. D. Utkina A. O. Rebouh N. Y. (2025). Precision agriculture for improving crop yield predictions: a literature review. Front. Agron. 7:1566201. doi: 10.3389/fagro.2025.1566201

[ref37] Sapra R. (2014). Using R2 with caution. Curr. Med. Res. Pract. 4, 130–134. doi: 10.1016/j.cmrp.2014.06.002

[ref38] Stevens T. Madani K. (2016). Future climate impacts on maize farming and food security in Malawi. Sci. Rep. 6:36241. doi: 10.1038/srep36241, 27824092 PMC5099946

[ref39] Taremwa C. Tumusiime S. Kyamanywa S. (2025). Prediction of maize yield in Uganda using CNN–LSTM architecture on multimodal climate and remote-sensing datasets. Artif. Intell. Agric. 9:100259. doi: 10.1016/j.aiia.2025.100259

[ref40] van Klompenburg T. Castaldi F. Srinivasan R. (2020). Review of machine learning applications in crop yield prediction. Comput. Electron. Agric. 177:105709. doi: 10.1016/j.compag.2020.105709

[ref41] Willmott C. Matsuura K. (2005). Advantages of the mean absolute error (MAE) over the root mean square error (RMSE) in assessing average model performance. Clim. Res. 30, 79–82. doi: 10.3354/cr030079

[ref42] Zhang H. Li Y. Chen R. (2025). Optimizing corn yield prediction: integrating multi-temporal UAS data and machine learning. Agric. Syst. 231:104971. doi: 10.1016/j.agsy.2025.104971

